# Draft Genome Sequences of Two Pyrazinamide-Resistant Clinical Isolates, *Mycobacterium tuberculosis* 13-4152 and 13-2459

**DOI:** 10.1128/genomeA.00758-15

**Published:** 2015-07-02

**Authors:** D. A. Maslov, K. V. Shur, O. B. Bekker, N. V. Zakharevich, M. V. Zaichikova, K. M. Klimina, T. G. Smirnova, Y. Zhang, L. N. Chernousova, V. N. Danilenko

**Affiliations:** a Laboratory of Bacterial Genetics, Vavilov Institute of General Genetics, Moscow, Russia; b Microbiology Department, Central Tuberculosis Research Institute RAMS, Moscow, Russia; c W. Harry Feinstone Department of Molecular Microbiology & Immunology, Department of Molecular Microbiology & Immunology, Bloomberg School of Public Health, Johns Hopkins University, Baltimore, Maryland, USA

## Abstract

We report draft genome sequences of two pyrazinamide (PZA)-resistant isolates, *Mycobacterium tuberculosis* 13-4152 and 13-2459. Isolate 13-4152 is PZA resistant, though it lacks mutations in known genes of PZA resistance. The comparative analysis of these genomes with those stored in GenBank revealed unique mutations, which may elucidate new mechanisms of PZA resistance.

## GENOME ANNOUNCEMENT

The persistence of *Mycobacterium tuberculosis* is a major problem facing tuberculosis control (1). Pyrazinamide (PZA) is a unique drug targeting persistent *M. tuberculosis* bacilli (2). PZA is a prodrug activated by pyrazinamidase (PZase) into its active form, pyrazinoic acid (POA) (3). Mutations in *pncA*, the gene encoding PZase, are the major mechanism of PZA resistance in *M. tuberculosis* (3–5). Recently, ribosomal protein S1 (RpsA), a key protein of trans-translation, which mediates a mechanism for saving stalled ribosomes or damaged mRNA, essential for dormant and persister cells (6, 7), was identified as a molecular target for POA (8), with mutations in it leading to PZA resistance (8, 9). Another gene involved in PZA resistance is *panD*, encoding an aspartate decarboxylase involved in pantothenate and coenzyme A biosynthesis (10).

For our analysis, we chose the *M. tuberculosis* isolate 13-4152 from an 11-year-old HIV-negative male patient with caseous pneumonia, and *M. tuberculosis* 13-2459, isolated from a 27-year-old HIV-negative male patient with infiltrative tuberculosis. The isolates were previously described by Maslov et al. (11). Both these isolates were resistant to PZA according to Bactec MGIT 960 DST, though 13-4152 lacked mutations in known PZA resistance genes (*pncA*, *rpsA*, and *panD*), while 13-2459 harbored a mutant *pncA* gene and had negative PZase activity. The genomic DNA from *M. tuberculosis* isolates 13-4152 and 13-2459 was purified by phenol-chloroform-isoamyl alcohol separation, followed by ethanol precipitation.

Genome sequencing was carried out on a Roche 454 GS Junior instrument (Roche, Switzerland), in the Laboratory of Bacterial Genetics, Vavilov Institute of General Genetics (Moscow, Russia). The generated reads were assembled to initial draft genomes using the GS De Novo Assembler (version 3.0; Roche). The automatic functional annotation results were obtained using the NCBI Prokaryotic Genome Annotation Pipeline (PGAAP) (http://www.ncbi.nlm.nih.gov/genomes/static/Pipeline.html).

A total of 150,356 reads were generated for *M. tuberculosis* 13-4152, assembled into a draft genome of 4,331,480 nucleotides (18-fold coverage, 151 contigs, 65.49% overall G+C content), with 4,004 predicted coding sequences, 45 tRNAs, and 3 rRNAs. The sequencing of *M. tuberculosis* 13-2459 generated 145,638 reads, which were assembled to an initial draft genome of 4,329,633 nucleotides (17-fold coverage, 167 contigs, 65.49% G+C content); 4,016 coding sequences, 45 tRNAs, and 3 rRNAs were predicted.

Based on housekeeping genes analysis (12) and the analysis of *oxcA* gene, these isolates were classified as representatives of the B0/W subgroup of the Beijing lineage. The comparative analysis of the genome sequences of *M. tuberculosis* isolates 13-4152, 13-2459, and the previously sequenced E186hv (13) allowed us to identify unique mutations in the *M. tuberculosis* 13-4152 genome. After mutations in PE/PPE genes and noncoding regions were excluded, a total of 15 nonsynonymous unique mutations in protein-coding genes were left: *rv0001* (*dnaA*), *rv0136* (*cyp138*), *rv0405* (*pks6*), *rv0668* (*rpoC*), *rv1032c* (*trcS*), *rv1349* (*irtB*), *rv1775*, *rv2006* (*otsB*), *rv2205c*, *rv2215* (*dlaT*), *rv2241* (*aceE*), *rv2752c*, *rv3383c* (*idsB*), *rv3384c* (*vapC46*), and *rv3634c* (*galE1*). Further functional analysis of these mutations is required to assess their potential role in mediating possible new mechanisms of PZA resistance in future studies.

### Nucleotide sequence accession numbers. 

These whole-genome shotgun projects have been deposited in GenBank under the accession numbers LAVG00000000 (*M. tuberculosis* 13-4152) and LDNL00000000 (*M. tuberculosis* 13-2459). The versions described in this paper are the first versions.
